# Upcycling discarded cellulosic surgical masks into catalytically active freestanding materials

**DOI:** 10.1007/s10570-022-04441-9

**Published:** 2022-02-01

**Authors:** Javier Reguera, Fangyuan Zheng, Ahmed Esmail Shalan, Erlantz Lizundia

**Affiliations:** 1 BCMaterials, Basque Center for Materials, Applications, and Nanostructures, UPV/EHU Science Park, 48940 Leioa, Spain; 2 Central Metallurgical Research and Development Institute (CMRDI), P.O. Box 87, Helwan, Cairo, Egypt; 3 Life Cycle Thinking Group, Department of Graphic Design and Engineering Projects, Faculty of Engineering in Bilbao, University of the Basque Country (UPV/EHU), 48013 Bilbao, Spain

**Keywords:** COVID-19 pandemic, Face masks, Non-woven cellulose, Upcycling, Photocatalysis, Nanozymes, Circular economy

## Abstract

**Abstract:**

The COVID-19 pandemic outbreak has resulted in the massive fabrication of disposable surgical masks. As the accumulation of discarded face masks represents a booming threat to the environment, here we propose a solution to reuse and upcycle surgical masks according to one of the cornerstones of the circular economy. Specifically, the non-woven cellulosic layer of the masks is used as an environmentally sustainable and highly porous solid support for the controlled deposition of catalytically active metal-oxide nanoparticles. The native cellulosic fibers from the surgical masks are decorated by titanium dioxide (TiO_2_), iron oxide (Fe_x_O_y_), and cobalt oxide (CoO_x_) nanoparticles following a simple and scalable approach. The abundant surface –OH groups of cellulose enable the controlled deposition of metal-oxide nanoparticles that are photocatalytically active or shown enzyme-mimetic activities. Importantly, the hydrophilic highly porous character of the cellulosic non-woven offers higher accessibility of the pollutant to the catalytically active surfaces and high retention in its interior. As a result, good catalytic activities with long-term stability and reusability are achieved. Additionally, developed free-standing hybrids avoid undesired media contamination effects originating from the release of nanoscale particles. The upcycling of discarded cellulosic materials, such as the ones of masks, into high-added-value catalytic materials, results an efficient approach to lessen the waste´s hazards of plastics while enhancing their functionality. Interestingly, this procedure can be extended to the upcycling of other systems (cellulosic or not), opening the path to greener manufacturing approaches of catalytic materials.

**Graphical abstract:**

A novel approach to upcycle discarded cellulosic surgical masks is proposed, providing a solution to reduce the undesired accumulation of discarded face masks originating from the COVID-19 pandemic. The non-woven cellulosic layer formed by fibers is used as solid support for the controlled deposition of catalytically active titanium dioxide (TiO_2_), iron oxide (Fe_x_O_y_), and cobalt oxide (CoO_x_) nanoparticles. Cellulosic porous materials are proven useful for the photocatalytic decomposition of organic dyes, while their peroxidase-like activity opens the door to advanced applications such as electrochemical sensors. The upcycling of cellulose nonwoven fabrics into value-added catalytic materials lessens the waste´s hazards of discarded materials while enhancing their functionality.
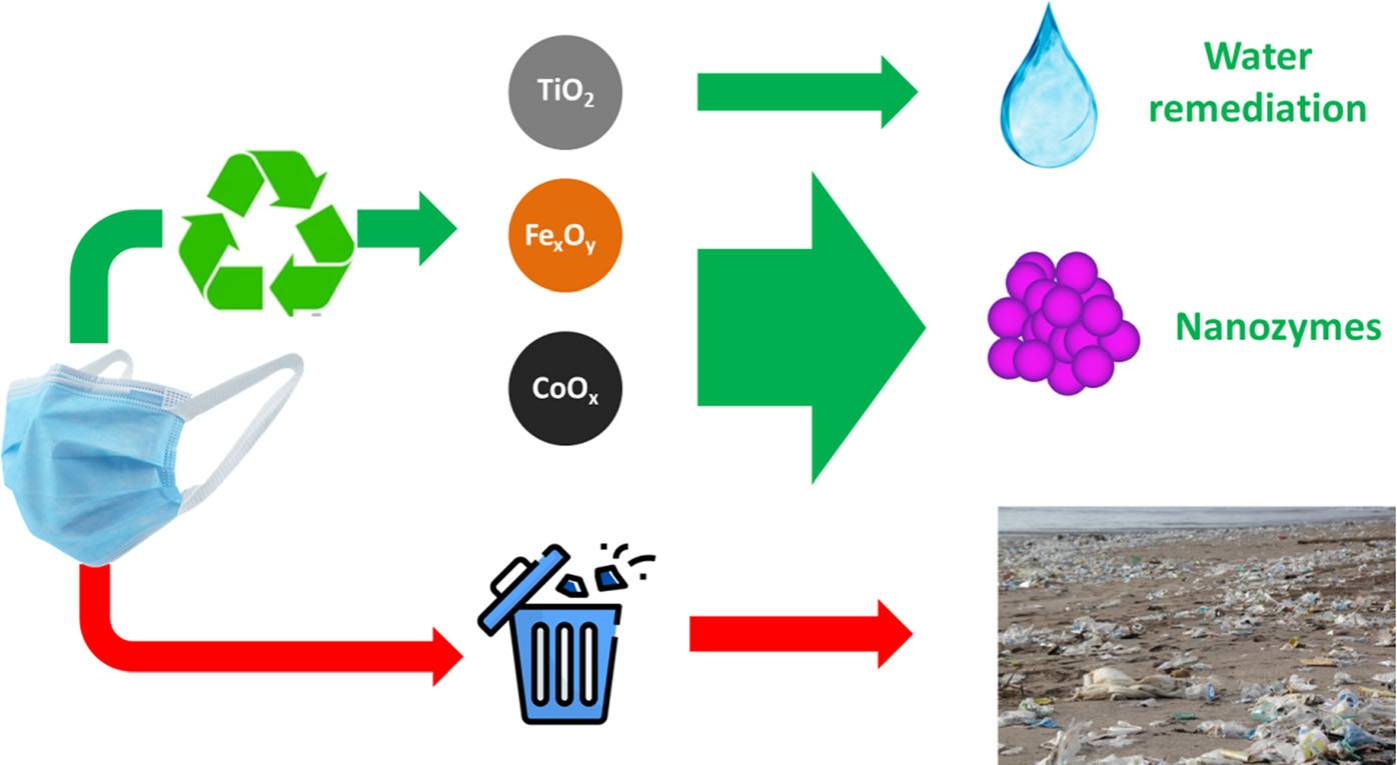

**Supplementary Information:**

The online version contains supplementary material available at 10.1007/s10570-022-04441-9.

## Introduction

Worldwide demand for surgical face masks has dramatically increased as many governments have made compulsory the use of face masks in public areas due to the COVID-19 pandemic outbreak (Feng et al. 2020). Face masks provide a physical barrier to reduce person-to-person virus transmission, which mostly originates from the respiratory microdroplets subsequent to sneezing and coughing (De Stefano et al. 2020). The face masks composed of polymeric materials have been specially useful to prevent the number of infectious viruses or bacteria in exhaled breath (Armentano et al. 2021). They are composed of a series of non-woven layers designed to avoid the passage of bacteria and viruses in both directions thanks to their tailored pore-geometry and large specific surface area able to block the viruses (Zhu et al. 2020).

The handling and disposal of the healthcare waste arising from the massive use of face masks have become a big concern, not only to control the spread of the SARS-CoV-2 virus (Thakur 2021), but also to lessen the environmental risks of medical waste when inappropriately disposed of (Wei et al. 2020). Discarded face masks are a booming threat to the planet as they have been encountered in the form of micro-plastics both in marine and landfill environments (Dharmaraj et al. 2021), reflecting the need to prevent such waste from uncontrolled leaking. In early 2022, 2 years after pandemic's start, the world is witnessing an explosion of new virus cases with the emergence of the Omicron variant (Kupferschmidt and Vogel 2022), leading to an accumulation of unused disposable face masks. Instead of representing a problem, these unused masks offer an opportunity for novel circular economy practices (Corrêa and Corrêa 2021; Selvaranjan et al. 2021). Interestingly, the surgical masks can be easily sterilized by dry heat, microwave radiation of ultraviolet light (UV-C light irradiation for 10 min from each side effectively kills the coronavirus) (Ben et al. 2021), or with 121 °C steam or H_2_O_2_ plasma (van Straten et al. 2021). As these approaches barely damage the functionality and the structure of the porous layers of the masks, these materials can be safely applied in different fields. Taking reuse and recycling practices into consideration as one of the cornerstones of circular economy (Kirchherr et al. 2017), providing second life to surgical face masks represents a plausible approach to lessen the environmental impacts arising from their undesired accumulation into marine and land environments. The upcycling of discarded materials into high-value functional products have been already proven efficient to reduce the waste´s hazards of polymers while enhancing their functionality (Górak et al. 2020; Lauria and Lizundia 2020).

Disposable surgical masks are typically made of three layers of non-woven fabrics (Armentano et al. 2021). The first layer prevents fluids carrier penetration, the second layer retains viruses and the inner third layer absorbs fluids from the user (Wibisono et al. 2020). Although the composition can vary, the filter layer typically consists of a cellulose-based fabric, while the outer layers are made of polypropylene or in many cases of cellulose layers, offering in this last case of a more skin-friendly surface (Bilgi et al. 2021). Provided by its abundant surface hydroxyl (–OH) groups, cellulose offers a recyclable and environmentally sustainable solid support for the controlled deposition of metal/metal-oxide nanoparticles (Mousli et al. 2020; Tang et al. 2020). Importantly, nano-, meso- and micro-porous membranes can be easily achieved with cellulose through diverse fabrication approaches (Lizundia et al. 2020), which makes this renewable material particularly versatile. The three -OH groups at the 2-, 3- and 6-position in the anhydroglucose repeating unit act as concomitant reducing and capping agents for metal/metal-oxide nanoparticle immobilization (Musino et al. 2021). Accordingly, different catalytically active nanoparticles have been deposited onto cellulosic substrates, including zinc oxide (Awan et al. 2018), palladium (Wu et al. 2016), platinum (Lizundia et al. 2019), or gold (Yan et al. 2016). The synergetic effects arising from the cellulosic substrate and the inherent catalytic activity of metal/metal-oxides open novel opportunities for a variety of applications.

In this framework, we focus our attention on water remediation given its pivotal role to support plant and animal life. The growing pressure arising from the world population increase coupled with the extended use of chemicals has resulted in contaminated water reservoirs (Steffen et al. 2015; Ccanccapa et al. 2016). Hence, organic pollutant removal from the water represents a global challenge for the twenty-first century and forms part of the 6th sustainable development goal (SDG) of the United Nations (Karpińska and Kotowska 2019). Photocatalysis results in one of the most promising approaches towards environmental remediation because it enables a platform to trigger diverse chemical reactions which decompose the pollutant into less-active and non-toxic species (Foteinis et al. 2018; Singh and Goldsmith 2020). In a similar fashion, it enables water disinfection through the degradation of pathogens such as bacteria or viruses (Foster et al. 2011; Zhu et al. 2018; Rodríguez-González et al. 2020). In comparison with other methods such as adsorption (Xia et al. 2020), filtration (Kondo et al. 2020), or reverse osmosis (Liu et al. 2015), photocatalyst particles can be reused with no need for reactivation and can be used under sun illumination to reduce the energetic cost. Originating from the inherent photocatalytic activity of titanium dioxide (TiO_2_) (Khin et al. 2012), the immobilization TiO_2_ nanoparticles onto a mechanically flexible solid support such as surgical masks enables a self-standing hybrid capable of catalyzing a large range of reactions when excited with external radiation. Additionally, undesired media contamination effects originating from the release of nanoscale particles are avoided. An additional benefit comes from the hydrophilic porous character of the cellulosic non-woven layer offering higher accessibility of the pollutant to the catalytically active surfaces (Marques et al. 2021). Importantly, photocatalytic sterilization is recognized as a sustainable, cheap, and effective disinfection approach for SARS-CoV-2, simplifying their use once discarded (Ghedini et al. 2021).

The application range of organic–inorganic hybrids can be expanded to a great extent replacing TiO_2_ with nanozymes, nanoscale-systems with intrinsic enzyme-like activities (Wang et al. 2018b; Liang and Yan 2019). Since the pioneering work by Scrimin et al. in 2004 (Manea et al. 2004), nanozymes have been attracting increasing attention for many applications including diagnostic medicine, biosensing, or environmental remediation (Meng et al. 2020). These artificial enzyme-like analogs are easy and cheap to manufacture, offer improved catalytic stability and remain stable under harsh physicochemical conditions in comparison with natural enzymes such as globular proteins (Jiang et al. 2019). Among the different nanoparticles that mimic the catalytic activity of enzymes such as peroxidase, oxidase, catalase, haloperoxidase, and others (Wei and Wang 2013; Liang and Yan 2019), Fe_3_O_4_ nanoparticles are specially attractive given their intrinsic ability to oxidize organic substances (Chen et al. 2012; Gao et al. 2017; Maharjan et al. 2020). After its discovery, other metal oxides such as CoO_x_ have shown similar activity (Mu et al. 2012; Guo et al. 2020), and opened the way to new types of enzyme-like activities.

Here we explore the potential of discarded surgical masks to develop novel heterogeneous catalytic materials. A general strategy for the fabrication of photocatalytically active or enzyme-mimetic active hybrid nanomaterials is shown upon embedding diverse catalytic metal oxide nanoparticles within the porous structure of cellulosic membranes. Well-known catalytically active materials including TiO_2_, Fe_x_O_y_, and CoO_x_ are used as model examples. The versatility of cellulose as a substrate for catalytically active materials results in materials with efficient degradation activities towards contaminants of emerging concern or marked peroxidase-like activities, paving the way for novel environmental cleanup systems, chemical processes, or sensing. Additional applications could arise in biomedicine or as antifouling fabrics and surfaces. In those cases, some concerns regarding biocompatibility (for instance with Co) or possible allergic effects may emerge. Remarkably, the upcycling of cellulosic surgical masks represents a step forward to face the massive mask disposal that occurred during the COVID-19 pandemic.

## Materials and methods

### Chemicals and materials

Surgical masks (EN14683, Type II) were kindly provided by Medline Industries, Inc., Australia. For the nanoparticle synthesis, cobalt acetate tetrahydrate was purchased at Alfa Aesar; ferric chloride (FeCl_3_, 99%), ferrous chloride tetrahydrate (FeCl_2_.4H_2_O, 98%), ethanediol, ethanol, 1,2-diaminoethane, hydrochloric acid (HCl 37%), Titanium (IV) isopropoxide (97%), ammonium hydroxide (NH_4_OH, 25–30% of ammonia) at Sigma-Aldrich; NaOH (98.0%-100.5%). For the catalysis experiments H_2_O_2_ (30% w/v) was purchased at Panreac; Methylene blue (MB), and 3,3',5,5'-tetramethylbenzidine (TMB), and acetate buffer (pH = 4.66) at Sigma Aldrich.

### Nanoparticle synthesis

#### *Synthesis of TiO*_*2*_* nanoparticles*

To synthesize TiO_2_ nanoparticles, Milli-Q water (200 mL) was added slowly into 10 g of titanium isopropoxide in a 400 mL beaker to generate the hydrolysis of the alkoxide and precipitation of hydrous titanium oxides. Immediately afterward, NaOH solution (1 M) was added slowly until pH reached 8. The mixed solution was kept thoroughly mixed by continuous stirring at 100 °C for 1 h. pH was checked again and readjusted with NaOH solution (1 M) to 8. Once the reaction was finished, the final solution was kept stirring at room temperature for 1 h. The white precipitate formed in the reaction was filtered and washed several times with deionized water. The last step was to dry the washed precipitate at 80 °C in an oven overnight and then anneal it at 400 °C for 3 h. The final product was ground with a pestle and mortar to obtain a fine powder.

#### *Synthesis of Fe*_*x*_*O*_*y*_* nanoparticles through co-precipitation (Fe*_*x*_*O*_*y*_* sample 1)*

Fe_x_O_y_ nanoparticles were prepared using a co-precipitation technique (Darezereshki 2010). FeCl_3_ and FeCl_2_.4H_2_O were dissolved in a 2 M HCl to form an aqueous solution with the concentration of 1 M for FeCl_3_ and 2 M for FeCl_2_.4H_2_O. After that, the ammonia solution (2 M) was dropped to this solution with vigorous stirring at room temperature for 2 h. The final pH was 9.5. The brown precipitate was then collected by filtration and rinsed three times with deionized water and ethanol. Finally, the washed precipitate was dried at 70 °C overnight.

#### *Synthesis of Fe*_*x*_*O*_*y*_* (Fe*_*x*_*O*_*y*_* sample 2) and CoO*_*x*_* through diol-based synthesis*

The precursors of the different metal oxides were prepared with cobalt acetate tetrahydrate (0.5 M) and a mixture of FeCl_3_ and FeCl_2_.4H_2_O as sources of CoO_x_, and Fe_x_O_y_, respectively. These different precursors were dissolved in an ethanediol solution containing 1,2-diaminoethane (1 M) stirred for at least 3 days to form a homogeneous precursor solution (Shalan et al. 2016). The solution was used as obtained without any further purification or calcination.

### Fabrication of cellulose-nanoparticle hybrid material

The chosen mask did not present any dye nor colorant, and comprised three non-woven cellulose layers (providing a face mask differential pressure of 24.71 Pa·cm^−2^), with a thicker interior layer and two identical and more porous exterior layers. The cellulose substrate was obtained from the exterior layers of a surgical mask providing two sheets of 15.5 × 17 cm^2^ after removing the mask borders. The cellulose sheet was immersed in the nanoparticle aqueous solution (50 mL) at a concentration of 1 g·L^−1^ and left for 3 h. After the incubation, the mask was rinsed and immersed in 50 mL of ultrapure water and left with mild shaking for 2 h. The supernatant was measured by UV–Vis and then discarded. The process was repeated several times until no trace of nanoparticles was observed by UV–Vis (4 times).

### Characterization

Attenuated total reflectance Fourier transform infrared spectroscopy (ATR-FTIR) measurements were performed on a Bruker Alpha FT-IR Spectrometer equipped with diamond ATR optics. Samples were dried at 50 °C overnight before characterization. 64 scans were taken in the range 400–4000 cm^−1^ with a resolution of 2 cm^−1^.

Nanoparticles were dispersed in water and drop-casted in a C-coated TEM grid and left to dry. Samples were measured in a JEOL 1400 working at 100 kV. Images were analyzed using Image J software package, and nanoparticle diameters were measured manually with more than 100 nanoparticles per sample.

Room temperature XRD has been conducted in a PANalytical Empyrean powder diffractometer in reflection mode using Cu-Kα radiation (*λ* = 1.5418 Å) operating at 45 kV and 40 mA to investigate the crystalline structure of synthesized P-dots. Samples were mounted on a zero background silicon sample holder. Data were collected from 5 to 80° 2*θ* (step size = 0.026º; angular resolution: 0.026º) at RT. Grain size was evaluated using Scherrrer’s formula, *τ* = *kλ/βcosθ* where the shape factor *k* was taken as 0.9, and *β* was obtained from the most intense peak after subtracting the instrumental FWHM of 0.1. The relative composition of the crystalline phases was obtained from the relative areas using a weighted average mass (Cano-Casanova et al. 2018). *W*_*A*_ = *K*_*A*_*I*_*A*_*/(K*_*A*_*I*_*A*_ + *K*_*B*_*I*_*B*_ + *I*_*R*_*)*, *W*_*B*_ = *K*_*B*_*I*_*B*_*/(K*_*A*_*I*_*A*_ + *K*_*B*_*I*_*B*_ + *I*_*R*_*)*, and *W*_*R*_ = *I*_*R*_*/(K*_*A*_*I*_*A*_ + *K*_*B*_*I*_*B*_ + *I*_*R*_*)*, where *W*_*A*_, *W*_*B*_, and *W*_*R*_ are the weight fractions of anatase, brookite, and rutile respective, *I*_*A.B, or R*_ denotes the integral area and *K*_*A*_ = 0.886 and *K*_*B*_ = 2.721 the correction coefficients.

Mechanical properties of the cellulose membranes were analyzed in uniaxial tensile testing mode on an AGS-X universal testing machine from Shimadzu equipped with a 500 N load cell in displacement control mode at a rate of 1 mm·min^−1^. Rectangle-shaped 70 ± 5 μm thick, 20 mm long, and 12 mm wide films were used.

For inductively coupled plasma (ICP) studies, the substrate was cut with a 1 × 1cm^2^ was introduced at the bottom of a 15 mL falcon tube, HCl and HNO_3_ (trace analysis purity) were added at a ratio of 3:1 to generate aqua regia in situ and left for 1 h. 5 mL of Milli-Q H_2_O was added and mixed and the solution was transferred to a new tube. This was repeated with another 5 mL of H_2_O, the sample was centrifuged and the supernatant was taken together with the previous solution to form a 10 mL solution to be analyzed by ICP. The samples were measured in a quadrupole mass spectrometer with an ICP source (Q-ICP-MS), model XSeries-II (Thermo). An internal standard was introduced by adding 100 µL of Yttrium at 500 ppb. The measured selected isotopes to perform the quantification were ^59^Co, ^57^Fe, and ^197^Au. The results showed a concentration of nanoparticles of 26.6, 90.5, 30.9, and 3.4 μg·cm^2^ for TiO_2_, Fe_x_O_y_ samples 1 and 2, and CoO_x_ respectively, which corresponds to 1.4, 4.2, 1.6, and 0.17 wt. % respectively (in terms of the metal atoms to the total substrate).

For XPS experiments, a drop of nanoparticle solution was deposited in the XPS holder and left to dry without further preparation. XPS measurements were performed in the SPECS instrument (Berlin, Germany) equipped with an analyzer Phoibos150 1D-DLD and monochromatic radiation source Al Kα (1486.7 eV). A first quick wide scan was carried out to determine the present elements (step energy 1 eV, dwell time 0.1 s, pass energy 80 eV) and then a detailed analysis was performed for the different elements (step energy 0.08 eV, dwell time 0.1 s, pass energy 30 eV) with an electron outgoing angle of 90°. The spectrometer was previously calibrated with Ag (Ag 3d5/2, 368.26 eV). The spectra were fit with the software CasaXPS 2.3.16, which models the Gauss-Lorentzian contributions after background subtraction (Shirley). Concentrations were calculated correcting the values with the relative atomic sensitivity factors (Scofield).

TiO_2_ nanoparticles were analyzed in a UV–Vis reflectance spectrometer (DRS), using a UV–Visible-NIR Jasco V-770 spectrometer equipped with a 150 mm diameter integrating sphere coated with Spectralon with 1 nm spectral resolution. DRS was carried out in the 250–2200 nm wavelength range. A Spectralon reference was used to measure the 100% reflectance and internal attenuators were used to determine zero reflectance in order to remove background and noise. The powders were placed on a white substrate, sealed, and mounted on a Teflon sample holder for the DRS measurement. The measured reflectance spectra were subsequently converted to Kubelka–Munk (K–M) absorption factors to evaluate the absorption spectra of the powders. The band-gap was estimated via the Tauc plot $\left[ {F\left( R \right)h\nu } \right]^{{\text{n}}} { }vs.h\nu$ where F(R) is the absorbance, *h* is the Planck constant, υ is the frequency, and n is the sample transition taken as *n* = 1/2) (Granqvist 1995; Sakthivel et al. 2006).

### Photocatalysis assays

A disk shape of the TiO_2_-containing substrate (*Ø* = 4 cm) was placed at the bottom of a beaker (100 mL), and then filled with 30 mL of methylene blue and left under mild orbital shaking. UV–Vis measurements were performed at different times to follow the absorption of the dye by the substrate. The photocatalytic reaction was produced by illumination the substrate from the bottom with a 10 W and 365 nm LED that produced 0.6 W optical power. The solution was maintained under mild shaking and samples were measured at different time intervals. UV–Vis spectra were acquired in a spectrometer (AvaSpec, Avantes) between 200 and 1100 nm. 1 mL of the solution was taken from the solution in a 1 cm optical path cuvette, was measured, and added again to the solution (the whole process took less than 30 s). The acquired spectra were analyzed using Spectragryph software package.

For the sake of comparison, the apparent reaction rate constant (*k*_*app*_, min^−1^) was calculated using a first-order kinetic following the Langmuir–Hinshelwood model according to the equation:

1 $$\ln \frac{C}{{C_{0} }} = - k_{app} \cdot t$$

where *C*_*0*_ represents the initial MB concentration (before catalyst incorporation into the polluted media) and *C* is the apparent concentration at time *t*.

### Peroxidase-like catalysis

A solution of 3',5,5'- tetramethylbenzidine (TMB) was prepared first dissolving the TMB in DMSO at a concentration of 1 mg·mL^−1^ and then diluting it in an acetate buffer (pH = 4.66) to obtain a final concentration of 0.1 mg·mL^−1^. The catalytic reaction was performed in a 4 mL glass cuvette. A piece of the substrate of 4.2 cm^2^ was weighed and added to the cuvette, then 3 mL of TMB solution was added and finally, 7.87 µL of H_2_O_2_ (30% vol.) was added to initiate the reaction. The reaction was followed in UV–Vis taking spectra at different time intervals. The reaction progress was monitored by representing the absorbance at 654 nm as a function of time. A kinetic analysis was performed using the degradation of H_2_O_2_ due to the oxidation of the substrate TMB, and fitting it to a first-order kinetic model.

## Results and discussion

### Scope of the work

**Scheme 1:**
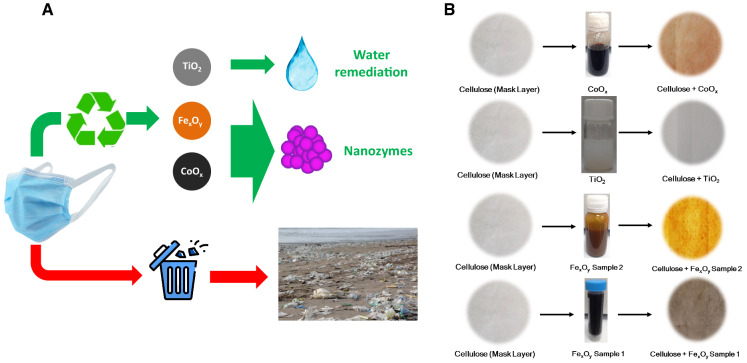
**A** Use of cellulosic surgical masks as a platform material to develop catalytic materials upon the incorporation of TiO_2_ nanoparticles and Fe_x_O_y_/CoO_x_ nanozymes. This procedure avoids the uncontrolled disposal of surgical masks. **B** Scheme with the preparation of catalytic substrates onto cellulose non-woven layers showing the macroscopic appearance of the masks before and after incubation with nanoparticle solutions (stored in the vial)

As summarized in Scheme 1A, here we aim to use the cellulosic layer within the surgical masks as a platform for the development of free-standing catalytically active materials. This approach faces some of the recognized limitations of inorganic nanoparticles when used for catalytic applications. When dispersed in a liquid media nanoparticles tend to aggregate and precipitate as a result of their high surface energy and surface change during the catalysis, thus the available active area to undergo reactions decreases dramatically (Grzelczak et al. 2010). Another equally relevant problem is the difficulty in removing dispersed catalysts from the reaction liquid which leads to undesired medium contamination. Finally, as dispersed nanoparticles are difficult to handle, their reuse is often compromised (Miceli et al. 2021). On the other hand, the cellulose substrates such as the ones found in masks can be reused into a high added value application, avoiding their uncontrolled disposal into marine or land environments (as floating marine debris, masks seriously impact the marine ecosystems) (Chowdhury et al. 2021). To explore the potential of surgical masks as three-dimensional substrates for catalytic applications, we turn our attention to TiO_2_ nanoparticles and Fe_x_O_y_ and CoO_x_ nanozymes to exploit their inherent water purification and peroxidase-like activity, respectively (Scheme 1B shows the macroscopic visual appearance of the masks before and after nanoparticle loading).

### Active material formation and substrate properties

**Fig. 1:**
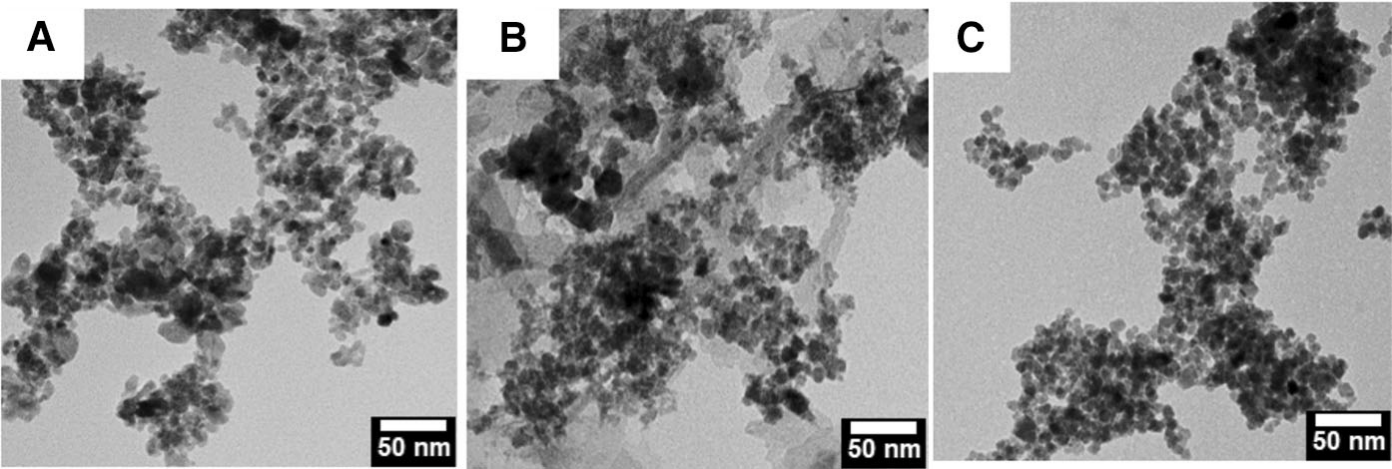
TEM images of synthesized nanoparticles: **A** TiO_2_ nanoparticles, **B** Fe_x_O_y_ sample 1, synthesized by co-precipitation, and **C** Fe_x_O_y_ sample 2 synthesized by the diol-based method

Several types of nanoparticles were synthesized as the active material to modify the mask substrates. This includes TiO_2_ as photocatalytic nanoparticles, two iron oxide (Fe_x_O_y_) nanoparticles synthesized by the coprecipitation method (sample 1) and the diol method (sample 2), and one cobalt oxide (CoO_x_) synthesized by the diol method. Figure 1A–C shows the TEM of the different nanoparticles, presenting an average size of 11.6 ± 4.2, 9.5 ± 3.3, and 8.0 ± 1.6 nm for TiO_2_, and Fe_x_O_y_ samples 1 and 2 respectively (see Fig. S1 for the size distribution plot). CoO_x_ nanoparticles were not visualized in TEM probably due to their very small size.

**Fig. 2:**
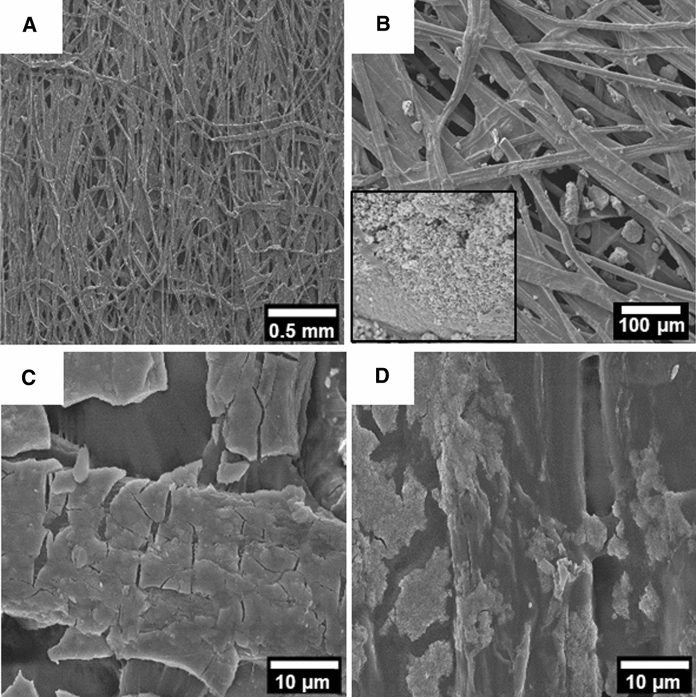
SEM images of the catalytic substrates. **A** non-woven cellulose obtained from the mask without any treatment. **B** Substrate loaded with TiO_2_ nanoparticles, and inset showing a zoom-in area completely coated with nanoparticles. **C** Substrate loaded with Fe_x_O_y_—sample 1 nanoparticles with high surface coating. D) Substrate loaded with Fe_x_O_y_—sample 2 nanoparticles exhibiting low surface coating

The exterior layers (inner and outer) of the masks showed a non-woven structure formed by randomly oriented filaments with an average thickness of 18.7 µm (Fig. S4) and a relatively smooth surface as shown in the SEM image of Fig. 2A. The nanoparticle-functionalized mask was obtained by incubating (impregnation process) the cellulose substrate in a concentrated solution of nanoparticles followed by a cleaning procedure to remove non-attached nanoparticles (see materials and methods). After incubation, a layer formed of islands of nanoparticles was appreciated on the sheet filament surfaces (Fig. 2B, D) with a homogenous distribution through the film (observed by SEM inspection in different regions and visually with a homogenous distribution of color). This good adsorption was expected, given the hydrophilic nature of the cellulose sheet and the large amount of hydroxyl groups. Nevertheless, highly different patterns were observed. For TiO_2_ The nanoparticles appeared in small aggregates intercalated in the fibers and small areas with highly covered fibers (Fig. 2B and inset respectively). For Fe_x_O_y_, the fibers showed a more homogeneous coverage but with different coating surface areas (Fig. 2C, D).

TiO_2_ nanoparticles were analyzed by XRD showing its crystalline structure corresponding mainly to anatase phase with a contribution of 65%, while the other phases were minority, rutile 23% and brookite 12% (Fig. S2). From the diffractogram and applying the Scherrer´s formula at the (100) peak, a crystalline grain size of 7.6 nm can be extracted, slightly lower than the one obtained by TEM and indicative of a high number of single crystal nanoparticles. Additionally, using diffuse reflectance spectrometry, a bandgap of 3.1 eV can be extracted (Fig. S3), a typical value for TiO_2_, which folds between the 3.05 and 3.2 eV for rutile and anatase phase respectively (Hanaor and Sorrell 2010; Sahu and Murty 2016).

**Fig. 3:**
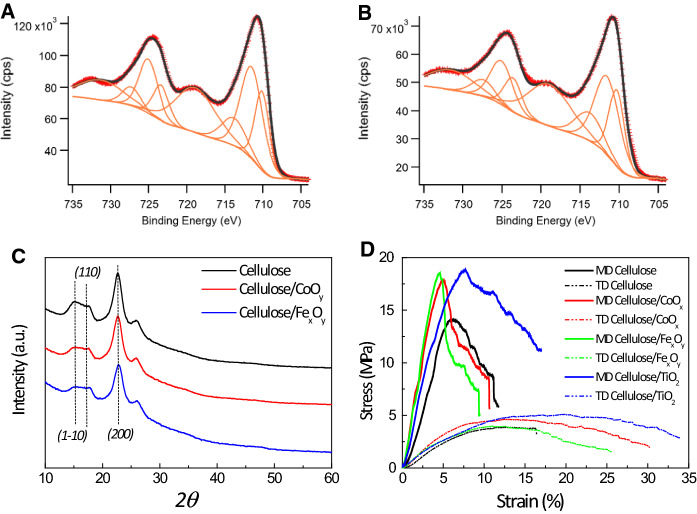
XPS spectra of Fe_x_O_y_ corresponding to the Fe 2p region for samples 1 (**A**) and 2 (**B**). Markers represent the measured spectrum, orange line the peaks to fit according to ref (Yamashita and Hayes 2008) and black solid line the fitting envelope. The Fe^3+^/Fe^2+^ obtained after the fitting corresponds to 2.13 for sample 1, and 1.95 for sample 2. In agreement with a majority of Fe_3_O_4_ phase. C) XRD patterns and D) representative tensile stress–strain curves of the samples. MD: machine direction; TD: transverse direction

To evaluate the crystalline phase of Fe_x_O_y_ and therefore their catalytic capabilities (Tokoro et al. 2018) the samples were checked by XPS (see Fig. 3A and B), and fitted either to only Fe^3+^ (for the case of Fe_2_O_3_) or both Fe^3+^ and Fe^2+^ (for the case of Fe_3_O_4_). The experimental data were fit in the Fe(2p) region of binding energies given a ratio of Fe^2+^/Fe^3+^ of 0.47 and 0.512. Note here, that despite the presence of a satellite peak in the 720 eV region, typically attributed to γ-Fe_2_O_3_, the envelope does not generate a good fit to the experimental data. Therefore, the ratio Fe^2+^/Fe^3+^, close to 0.5, and its better fit suggest a majority of Fe_3_O_4_ crystalline phase, which should show higher activity than other crystalline phases (Chen et al. 2012). Finally, the solution containing CoO_x_ didn´t show any crystalline peak in XRD, probably due to its difficult purification and high presence of partially reacted reagents. XRD was conducted to confirm whether or not changes in the crystalline structure of cellulosic support took place during the metal oxide nanoparticle decoration process. As shown in Fig. 3C, neat cellulose was characterized by three wide diffraction peaks centered at 2*θ* = 15.1, 17.5, and 22.7° arising from the *(1–10)*, *(110)*, and *(200)* crystal planes of cellulose I (JCPDS card no. 00–050-2241), respectively (Xu et al. 2013). The XRD pattern of metal-oxide decorated samples was also dominated by those characteristic reflections, indicating that the sample preparation process was mild enough to maintain the crystalline structure of the cellulosic support unchanged. The fact that no marked new diffraction peaks were observed suggests a low concentration of deposited metal oxide nanoparticles.

Mechanical properties of synthesized membranes were determined under uniaxial tensile testing mode to predict the capacity of such hybrid materials to withstand external stresses under application as catalysts. During fabrication, cellulose fabric is submitted to a stretching process in two orthogonal directions, producing biaxially oriented fabrics with anisotropic structural and mechanical properties (Lizundia et al. 2016; Kröling et al. 2018). Therefore, Fig. 3D summarizes obtained representative tensile stress–strain curves for the two orthogonal directions; machine direction (MD) which correlates with the stretching direction during cellulosic fabric preparation, and its transverse direction (TD). To enable an accurate comparison, Table 1 displays the main average and standard deviation mechanical property values in the MD direction, including Young´s modulus (*E*), elongation and stress at yield (*ε*_*y*_, *σ*_*y*_) and the elongation and stress at break (*ε*_*b*_, *σ*_*b*_). Neat cellulosic fabric shows a semiductile behavior with Young´s modulus of 339 ± 88 MPa, *σ*_*y*_ of 14.3 ± 1.1 MPa and an elongation at break of 11.0 ± 0.8% (the modulus decreases to 51 MPa while *ε*_*b*_ reaches ~ 18% in the TD). Obtained *E* is comparable to soft polyesters (*E*: 0.3–1 GPa) (Ribeiro et al. 2021), or natural materials such as leather or wood (*E*: 0.1–1 GPa) (AL-Oqla and Salit 2017), which ensures an adequate mechanical adaptability of the membranes when in use. Overall, metal oxide nanoparticle decoration increases both Young’s modulus and tensile strength up to 550–570 MPa and 17.8–18.1 MPa, respectively, suggesting a mechanically reinforcing effect of inorganic nanoparticles. In spite of the stiffening effect provided by inorganic nanoparticles, the ductility remains barely unchanged, keeping the elongation at break above 10%. Importantly, achieved *ε*_*b*_ values are larger than results obtained for other porous materials such as Celgard 2400 (based on a petroleum-derived polymer) or glass microfiber filters, with elongations at break of 3 and 5.8%, respectively (Tian et al. 2019; Gonçalves et al. 2019). Such adequate ductility ensures that the membranes will not break apart when applied as catalytically active free-standing hybrid materials.

**Table 1:** Main representative parameters of tensile test for cellulose/metal oxide nanoparticle membranes. *E*: Young´s modulus; ε_y_: elongation at yield; σ_y_: stress at yield; ε_b_: elongation at break; σ_b_: stress at break

	*E* (MPa)	*ε*_*Y*_ (%)	*σ*_*y*_ (MPa)	*ε*_*b*_ (%)	*σ*_*b*_ (MPa)
Neat cellulose	340 ± 90	6.4 ± 1.3	14.3 ± 1.1	11.0 ± 0.8	8.6 ± 0.7
Cellulose/CoO_y_	550 ± 100	5.1 ± 0.2	17.8 ± 1.0	10.8 ± 1.1	9.1 ± 1.5
Cellulose/Fe_x_O_y_	570 ± 70	4.5 ± 0.1	18.1 ± 1.5	9.9 ± 0.6	7.8 ± 0.4
Cellulose/TiO_2_	430 ± 70	7.8 ± 0.2	18.9 ± 4.2	17.3 ± 3.3	8.9 ± 0.4

### Photocatalytic performance for the degradation of dyes

**Fig. 4:**
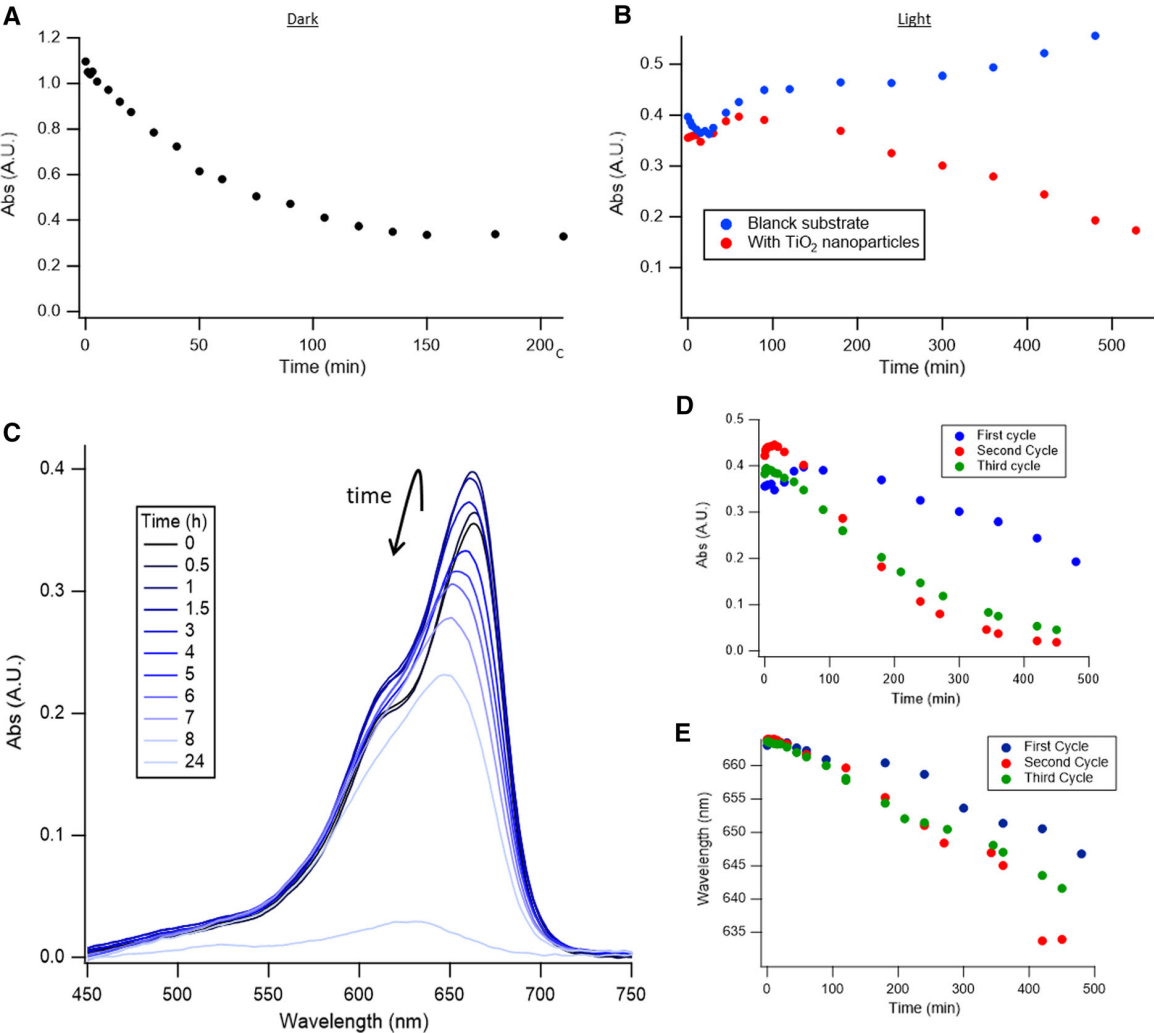
Photocatalytic degradation of methylene blue by TiO_2_-modified cellulose substrates. The extent of catalysis was analyzed by following the maximum absorbance of methylene blue (664 nm). (**A**) Variation of absorbance of methylene blue in illumination absence. (**B**) Variation of absorbance under UV-illumination for substrates with (red) and without (blue) TiO_2_ nanoparticles. (**C**) Spectra of the methylene blue solution at different illumination times, showing an initial increase and a posterior decrease of absorbance. (**D**) and (**E**) Absorbance and wavelength at the maximum respectively in a 3 cycles reusability test

The photocatalytic properties of the TiO_2_-functionalized mask were assayed in the degradation of the dye methylene blue. The initial absorption (in dark) of methylene blue and the subsequent photocatalytic degradation (under UV light) was monitored by UV–Vis spectroscopy following the methylene blue main peak at 664 nm corresponding to the mesomer II (Fernández-Pérez and Marbán 2020). Figure 4A shows the decrease of solution absorbance due to the absorption of methylene blue by the cellulose substrate when the substrate was immersed in its solution. The cellulosic natural porous structure together with its inherently hydrophilic nature provided by the many –OH groups induced a considerable absorption of the dye that accounted for more than 60%. This marked absorption favors the posterior catalysis by bringing the pollutant to the region where photocatalytic nanoparticles are located. The absorption process was finished between 2 and 3 h after the immersion of the substrate (Fig. 3A), and no further changes were observed even after long periods (more than 1 day). Once the absorption was finished the sample was exposed to UV-light with a light power of 50 mW·cm^−2^ which promotes the generation of electron–hole pairs that migrate to the surface of the nanoparticle and generate the simultaneous reduction of oxygen and oxidation of water generating reactive species that degrade the organic pollutants close to their surface (Zangeneh et al. 2015; Schreck and Niederberger 2019). The formation of high levels of reactive oxygen species (ROS) during UV illumination of filter paper/TiO_2_ nanowires has been recently observed by Horváth et al. (2020). These ROS are capable of degrading pollutants, including MB.

Figure 4B (in red) shows the decrease in absorbance due to the photocatalytic degradation of the dye. Interestingly the absorbance exhibits an initial increase, which is attributed to light-induced desorption and has been previously observed in TiO_2_ photocatalysis (Xu et al. 2014). In fact, when a control substrate is used (Fig. 4B, in blue), a continuous increase in absorbance is observed, indicating that light-induced desorption is produced at the cellulose matrix. For long times (1 day), the reaction was complete for the TiO_2_-containing substrate (A ≈ 0.01), while for the blank substrate only a small decrease of absorbance was observed (A ≈ 0.3, compared with the 0.4 that appeared at the beginning of the illumination) due to methylene blue photobleaching. Together with the decrease of absorbance in the photocatalysis, there was also a concomitant blue-shift of the main peak by 20 nm after 8 h of catalysis, (see corresponding UV–Vis spectra in Fig. 4C) indicating chemical changes produced in the degradation of the molecule

The Langmuir–Hinshelwood kinetic model has proven to be useful to describe the kinetics of photocatalytic reactions of pollutants in aqueous systems (Petukhov 1997; Yonar et al. 2006; Chiou et al. 2008). According to Fig. S5 and Eq. 1, a *k*_*app*_ value of 2.6 × 10^–3^·min^−1^ is obtained for the first cycle. As summarized in Table 2, this conversion rate is above the *k*_*app*_ reported for other related systems, with values of 1.1 × 10^–3^·min^−1^ for TiO_2_-nanoparticles immobilized within a cellulose nanofibre monolith (MO decomposition) (Lucchini et al. 2018), or 0.9 × 10^–3^·min^−1^ for TiO_2_-nanoparticles immobilized onto a macroporous SiO_2_ matrix (MO decomposition) (Marques et al. 2021). Although larger *k* values can be obtained by dispersion TiO_2_ nanoparticles into the pollutant (71 × 10^–3^·min^−1^ for MB under 254 nm irradiation (Chen and Hsu 2021). 18 × 10^–3^·min^−1^ for MB under 365 nm irradiation (Azeez et al. 2018), or 2.8 × 10^–3^·min^−1^ for MO under UV irradiation) (Zheng et al. 2019). the practical implementation of dispersed catalytically active nanoparticles bears serious limitations due to their difficult removal from the media.

**Table 2:** Comparison of different photocatalysts and the obtained apparent rate constant (*k*). MB: methylene blue; MO: methyl orange

Photocatalyst	Pollutant	Dispersed/immobilized	Radiation (λ, hν)	*k* (min^−1^)	References
TiO_2_	MB	Immobilized (cellulose non-woven)	365 nm (400 W·m^−2^)	2.6–8.2 × 10^–3^	This work
TiO_2_ (non-aqueous sol–gel)	MO	Immobilized (macroporous SiO_2_)	Solar simulator, 1 sun (1000 W·m^−2^)	0.9 × 10^–3^	(Marques et al. 2021)
TiO_2_ (non-aqueous sol–gel)	MO	Immobilized (cellulose nanofibre monolith)	Solar simulator, 1 sun (1000 W·m^−2^)	1.1 × 10^–3^	(Lucchini et al. 2018)
TiO_2_ (P25)	MB	Dispersed (unknown concentration)	254 nm (36 W)	71 × 10^–3^	(Chen and Hsu 2021)
TiO_2_ (non-aqueous sol–gel)	MB	Dispersed (100 mg·L^−1^)	365 nm (6 W)	18 × 10^–3^	(Azeez et al. 2018)
TiO_2_ (non-aqueous sol–gel)	MO	Dispersed (500 mg·L^−1^)	Solar illumination	2.8 × 10^–3^	(Zheng et al. 2019)
TiO_2_ (solvethermal-calcination)	MO	Dispersed (500 mg·L^−1^)	Visible light (300 W)	1.6 × 10^–3^	(Wang et al. 2018a)

To study the reusability of the substrate, the same substrate was applied in two more photocatalytic degradation cycles. The substrate produced the same amount of absorption (in dark) in all experiments. Under illumination, however, the photocatalytic performance improved after the second cycle (see Fig. 4D and E for peak absorbance and wavelength at the maximum respectively). Similar performance to the second cycle was produced during the third cycle, indicating good reusability of the substrate (*k*_*app*_ of 8.2 and 5.2 × 10^–3^·min^−1^ for the second and third cycles, respectively). The dissimilar behavior produced in the first cycle can be rationalized due to a photocatalytic cleaning of the surface of the TiO_2_ nanoparticles and removal of sub-products generated during the synthesis and processing steps (McGuinness et al. 2016; Adachi et al. 2018). Importantly, the chemical structure of the cellulose substrate was not damaged or altered after photocatalytic reactions as the fingerprint FTIR spectrum of cellulose (broad O–H vibration band at 3600–3200 cm^−1^, narrow C–H band at 2902 cm^−1^ or the C–O–C bending at 1160 cm^−1^) remained unchanged after methylene blue soaking and the 3 photocatalytic cycles (Fig. S6) (Nguyen et al. 2018). Altogether, results suggest good reusability of the catalytic materials as neither activity decrease nor substrate degradation occurs.

### Peroxidase-like catalysis

**Fig. 5:**
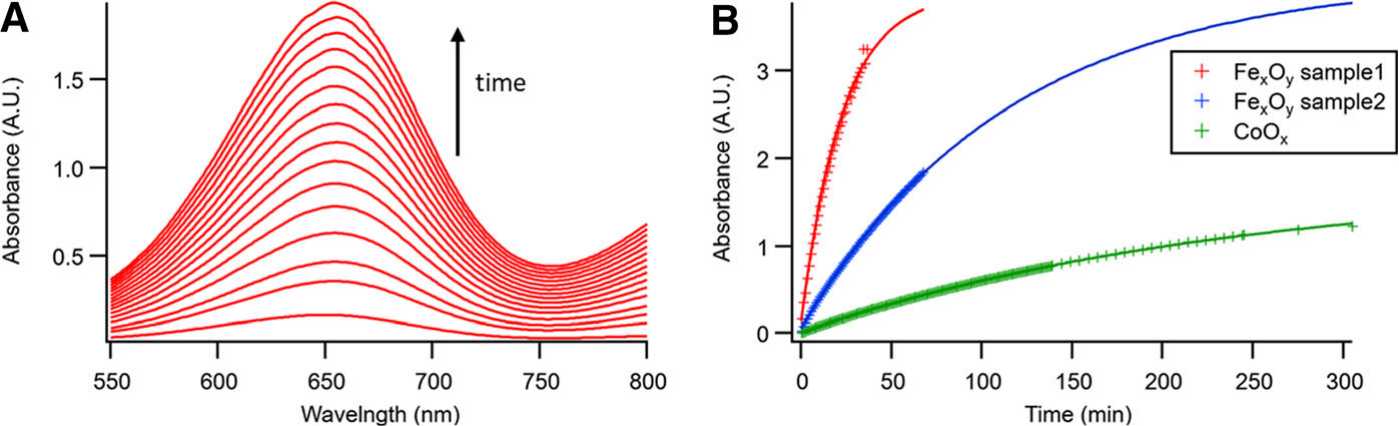
Peroxidase-like catalytic kinetic of different cellulose-functionalized substrates with nanoparticles of Fe_x_O_y_ (samples 1 and 2) and CoO_x_. **A** Spectra of the reaction at different time intervals showing the appearance of an intense band corresponding to the oxidation of TMB by H_2_O_2_ catalyzed by the substrate. **B** Absorbance at 664 nm as a function of time for the three different substrates

A Section (4 cm^2^) of the catalytic cellulose substrate was introduced in a glass cuvette containing a solution of TMB at pH = 4.66, and then a small quantity of H_2_O_2_ was added to start the reaction. The solution changed from transparent to blue due to the appearance of a peak at 654 nm (Fig. 5A) of the generated oxidized TMB. The three substrates showed a clear catalytic effect as summarized in Fig. 5B (see the fitting in Fig. S7). Considering the H_2_O_2_ degradation, we extrapolated the peroxidase-like catalytic activity rate of fabricated nanozymes. Values of *k* = 5.6 × 10^–2^ min^−1^, 1.2 × 10^–2^ min^−1^, and 1.8 × 10^–3^ min^−1^ are achieved for Fe_x_O_y_ (sample 1), Fe_x_O_y_ (sample 2) and CoO_x_, respectively, showing a peroxidase-like catalytic kinetic constant for Fe_x_O_y_ one order-of-magnitude higher than CoO_x_ for the TMB oxidation when H_2_O_2_ acts as oxidant. An interesting advantage of developed materials over other cellulose-supported nanohybrids is their easy of fabrication in comparison with more complex systems relying on multicomponent-materials (Hou et al. 2021), which show a robust peroxidase-like activity but are complex to fabricate.

As mentioned above, both sample 1 and sample 2 the Fe_x_O_y_ showed a similar composition corresponding mainly to the magnetite phase. Furthermore, sample 2 showed a slightly smaller size, 8.0 instead of 9.5 nm, which should favor its catalytic performance given its larger surface area. ICP analysis of the three different substrates was then performed to account for the possible concentration differences. The ICP showed concentrations, in terms of metal element per substrate area, of 90.5, 30.9, and 3.4 μg·cm^2^ which corresponds to 4.2, 1.6, and 0.17% for Fe_x_O_y_ sample 1 and 2, and CoO_x_ respectively. This was in agreement with what was visually observed by SEM (Fig. 2), where high coverage was observed for Fe_x_O_y_ sample 1, nanoparticle islands for Fe_x_O_y_ sample 2, and barely any nanoparticle for CoO_x_. The high difference in nanoparticle load seems to be the main mechanism for the notable differences in catalytic performance. The different chemical nature or type of nanoparticle synthesis is therefore affecting how the nanoparticles are absorbed in the cellulose substrate and therefore it is the main responsible for the efficiency of the nanozyme-based catalytic substrates. Interestingly, the cellulose/Fe_x_O_y_ samples could be also applied in additional catalytic reactions, such as those aimed for organic synthesis (Kamel and Khattab 2021).

Developed materials do not only show efficient activities towards the degradation of contaminants of emerging concern or marked peroxidase-like activities, but rely on well-known biocompatible nanomaterials. In fact, iron oxide and TiO_2_ are FDA-approved for use as pharmaceutical and food additives. Precisely, iron oxide nanoparticles, widely used in biomedical applications and show a relatively low cytotoxic effect, while TiO_2_ nanoparticles are broadly used in many consumables such as sunscreens, food additives or coatings (Yildirimer et al. 2011). However, some adverse cytotoxic effects have been reported for cobalt oxide, which can release Co ions that can result in generating oxidative stress (Cavallo et al. 2015). The biocompatibility of used metal oxide nanoparticles coupled with the innocuous character of cellulose makes these materials interesting for biomedical applications, although special care should be paid to the possible allergic reactions against human skin (Larsen et al. 2010; Cho et al. 2012; Horie et al. 2015; Ngobili and Daniele 2016; Lim et al. 2021).

## Conclusions

The aim of this work was to develop catalytically active free-standing materials using porous cellulosic substrates that originate from waste. Accordingly, after the incorporation of TiO_2_, CoO_x_, and Fe_x_O_y_ nanoparticles onto the surface of the cellulose non-woven layer of surgical masks, materials with proven catalytic activity were obtained. Microscopic observations revealed a homogeneous coating of inorganic nanoparticles onto the three-dimensional porous structure of the cellulosic membranes by a simple and implantable method. A photo-initiated decomposition of organic pollutants from an aqueous solution was demonstrated by the cellulosic mask/TiO_2_ system, which effectively decomposes methylene blue under UV illumination. Additionally, the cellulosic mask/Fe_x_O_y_ system demonstrated a good peroxidase-like activity, opening the door to advanced applications such as electrochemical sensors for hydrogen peroxide. In comparison with previous works based on dispersed nanoparticles, this approach has the advantage that relies on catalytically active nanoparticles attached onto a hydrophilic free-standing porous cellulosic substrate. This feature avoids nanoparticle aggregation during the catalytic reactions, circumvents medium contamination issues arising from nanoparticle release effects, and enables an easy handling of the material and its subsequent reuse for several cycles. The synthetic procedure reported here holds great potential for the development of catalytically active materials by a greener-fabrication method as it can be easily extended to the upcycling of other related systems, either cellulosic or not. This is particularly relevant given the large amount of accumulated polymeric waste and the widespread efforts in the burgeoning area of catalysis for environmental applications.

## Supplementary Information

Below is the link to the electronic supplementary material.

Supplementary file1 (DOCX 311 KB)

## Data Availability

All the data used to support the findings of this study are included within the article.
