# Supplementary material for: Upcycling discarded cellulosic surgical masks into catalytically active freestanding materials
Source: Cellulose (Lond). 2022 Feb 1;29(4):2223–40. doi: 10.1007/s10570-022-04441-9 (PMC8805669; doi:10.1007/s10570-022-04441-9)
Supplement: Supplementary file 1 — Supplementary file1 (DOCX 311 KB) [file 10570_2022_4441_MOESM1_ESM.docx]

Electronic Supplementary Information (ESI)

**Upcycling discarded cellulosic surgical masks into catalytically active freestanding materials**

*Javier Reguera^a*^,* [*Fangyuan Zheng*](https://www.researchgate.net/scientific-contributions/Fangyuan-Zheng-2192773216)*^a^, Ahmed Esmail Shalan^a,b^, Erlantz Lizundia^a,c,^*^*^

^a^ BCMaterials, Basque Center for Materials, Applications, and Nanostructures, UPV/EHU Science Park, 48940 Leioa, Spain.

^b^ Central Metallurgical Research and Development Institute (CMRDI), P.O. Box 87, Helwan, Cairo, Egypt.

^c^ Life Cycle Thinking Group, Department of Graphic Design and Engineering Projects, Faculty of Engineering in Bilbao. University of the Basque Country (UPV/EHU), Bilbao 48013, Spain.

^*^Corresponding authors: javier.reguera@bcmaterials.net; erlantz.liizundia@ehu.eus

**
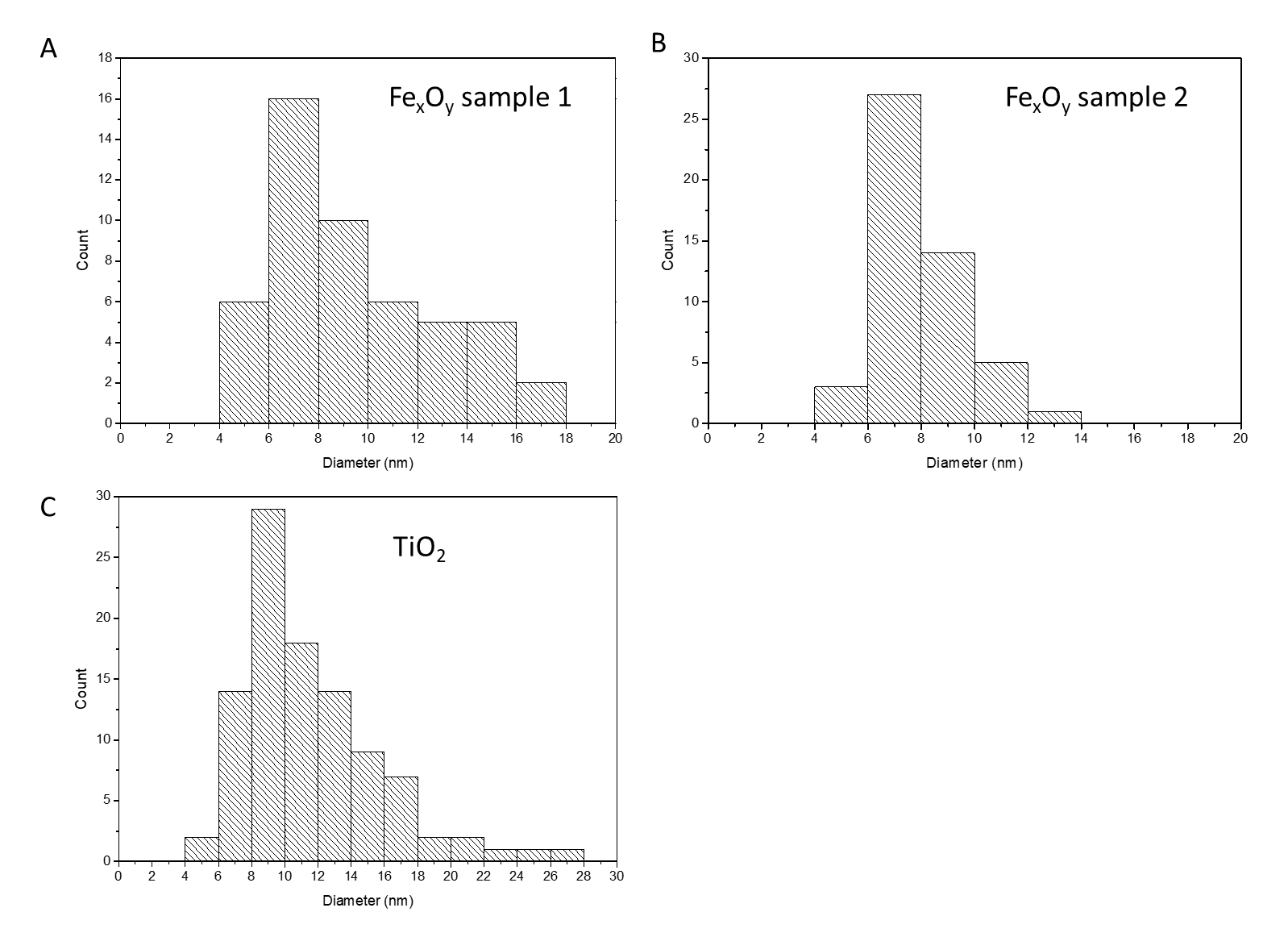
**

**Fig. S1.** Nanoparticle size distribution for the different nanoparticles: A) Fe_x_O_y_ sample 1 shows an average size of 9.5 ± 3.3 nm. B) Fe_x_O_y_ sample 2 shows an average size of 8.0 ± 1.6 nm. C) TiO_2_ shows an average size of 11.6 ± 4.2 nm.

**Fig. S2.** XRD diffractogram of TiO_2_ nanoparticles, and identification of the different peaks ascribed to the TiO_2_ three different phases, Anatase, Rutile, Brookite.

**Fig. S3.** Diffuse reflectance of TiO_2_ nanoparticles, and bandgap calculation after line fitting in the linear region (approx. 3.2 - 3.4 eV), E_g_ = 3.1 eV.

**Fig. S4.** Fiber thickness distribution of the non-woven cellulose layer of a face mask. The average thickness is $\bar{t}$ = 18.7 ± 8.0 μm.

**Fig. S5.** Photocatalytic activity of the mask/TiO_2_ system for the decomposition of methyl blue during three successive cycles. The linear fitting was performed in the linear region after the adsorption/desorption process was finished.

**Fig. S6.** A) FTIR spectra of the neat cellulosic mask, the mask after TiO_2_ loading, the cellulose/TiO_2_ mask after being soaked into methylene blue, and the cellulose/TiO_2_ mask after photocatalysis reaction. B) panel shows a magnified view of the 1800-800 cm^-1^ region.

**Fig. S7.** Catalytic activity of the peroxidase-like nanozyme-based substrates (mask/Fe_3_O_4_ and mask/CoO_x_ systems). Kinetics of the degradation of H_2_O_2_ by the oxidation of 3,3',5,5'-tetramethylbenzidine (TMB).
